# Environmental Impact of Synthetic Cell Technology: Review of Life Cycle Assessment Data for Feedstocks and Production Methods

**DOI:** 10.1002/cbic.70463

**Published:** 2026-07-30

**Authors:** Christina Hopf, Mehdi Ravandeh, Jan Steinkühler

**Affiliations:** ^1^ Bio‐Inspired Computation Institute of Electrical and Information Engineering Kiel University Kiel Germany; ^2^ Kiel Nano, Surface and Interface Science KiNSIS Kiel University Kiel Germany

## Abstract

Bottom‐up synthetic cells are frequently framed as enabling technologies for a future green bioeconomy, yet their environmental impacts remain poorly quantified. Here, we review and synthesize available life cycle assessment (LCA) data for the feedstocks, production routes, and assembly methods commonly used in synthetic cell research, focusing on *cradle‐to‐gate* system boundaries. We consider lipids from plant and algal sources, amphiphilic diblock copolymers, recombinant proteins, crude and PURE (protein synthesis using recombinant elements) cell‐free protein synthesis (CFPS) systems, and key assembly approaches including bulk emulsification and microfluidics. We argue that many of the identified components may also play a role in future generations of synthetic cells that undergo primitive autonomous growth and cell cycles. The data illustrate how design choices in compartment composition, encapsulated biochemistry, and assembly efficiency can shift impacts by orders of magnitude. Early integration of LCA‐informed design, such as favoring lower purity where functionally acceptable, using shared feedstocks, reducing material excess, and employing alternative autotrophic or solvent‐free production routes, will be decisive for achieving environmentally viable synthetic cell technologies.

## Graphical abstract

Synthetic cells are positioned as enablers of green technology. But what is known about the environmental impact of synthetic cell production? In this review, we examine drivers of greenhouse gas emissions, toxicity, and other environmental impact categories of synthetic cell components. We identify knowledge gaps and promising directions that unlock the full potential of this emerging field for green transformation.

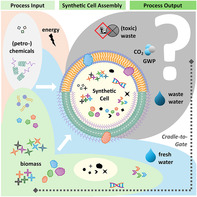

## 1 Introduction

**FIGURE 1:**
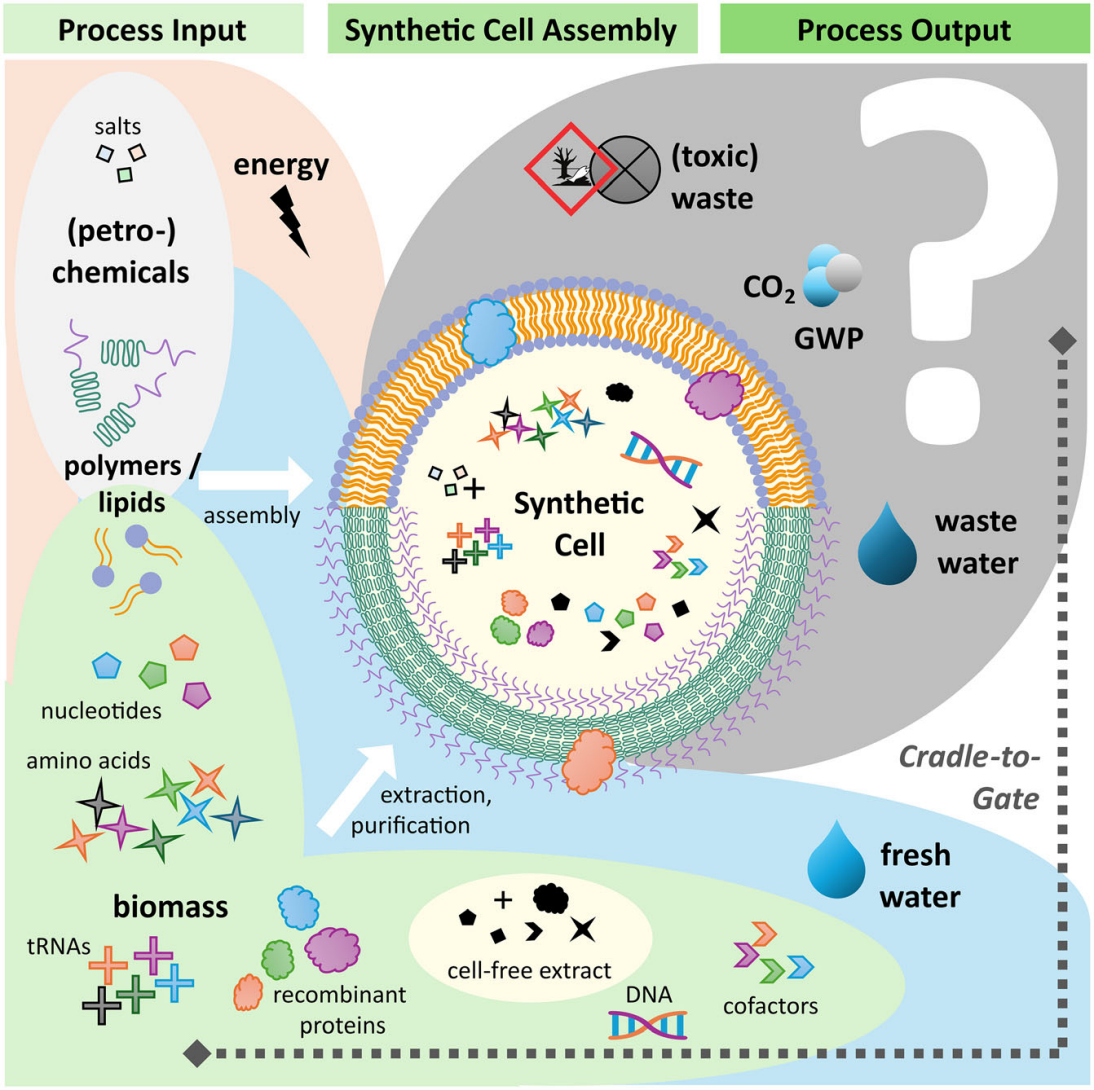
Overview of different contributions to the life cycle of synthetic cell manufacturing.

Many projected applications of bottom‐up synthetic cells align with the goals of the “green bioeconomy” [1]. A central aim is to reduce waste generation, energy consumption, and, in particular, CO_2_ emissions. Because circular processes in nature are associated with carbon neutrality and recyclability, these positive traits are sometimes implicitly transferred to “synthetic cells” and to biotechnology more broadly. However, it is well recognized that biotechnology processes do produce emissions to air, water, and soil, potentially leading to climate change, ozone depletion, resource depletion, acidification, biodiversity loss, and other impacts [2]. One way to assess impact is the life cycle assessment (LCA) method, which evaluates a product's environmental impact within a system boundary. In this review, we examine LCA studies on the feedstocks and production methods relevant to the synthetic cell assembly. By this, we aim to highlight the impact of choices in synthetic cell formation and identify gaps that require further research (Figure 1).

One might object that truly autonomous synthetic cells have not yet been realized, making a review of their environmental impacts premature. For this review, a practical working synonym for the synthetic cells investigated here is multifunctional liposomes (and closely related polymersomes), which already see widespread use in applications across diverse sectors, including food production, biofuels, plastic degradation, carbon capture, and environmental monitoring [3, 4]. In addition, not every biological cell is capable of autonomous replication, and the first generation of synthetic cells may similarly rely on the addition of purified components to sustain function. In the synthetic cell community, there is currently little discussion of the environmental impact such systems would have. Given the wide range of applications that even these, so far, incomplete synthetic cells are poised to address, we see clear value in identifying important environmental aspects of their production early. Indeed, LCA should already be considered during early development of all production technologies, ensuring that future production processes are designed to be sustainable from the start [5]. Apart from potential future applications, there are also attempts to reduce the environmental impact of laboratory research itself, and the data reported here could guide such initiatives. Finally, this review also serves as a primer for the synthetic cell community on key concepts and terminology in environmental impact assessment (see Table 1).

**TABLE 1:** Terminology used in this review.

**•** Life cycle assessment (LCA): A method for quantifying environmental impacts across the life cycle of a process by parameterizing material and energy inputs and outputs within a defined system boundary and translating them into environmental impact indicators. Impacts are typically assessed from raw material extraction and processing (“cradle”) using life cycle inventory (LCI) databases, which include both direct process inputs and indirect inputs such as energy and upstream supply chain requirements.
**•** System boundary: *Cradle*‐*to*‐*gate*, from feedstock production to ready‐to‐use synthetic cell formulations exiting a hypothetical factory. *Cradle‐to‐grave* would include the life cycle of a product after its usage.
**•** Functional unit: Quantified description of the function or service provided by the system under study, used as the reference to which all inputs and outputs are normalized (e.g., “1 kg of dry synthetic cell formulation,” “1 L of ready‐to‐use medium,” or “one experimental run”).
**•** Global warming potential (GWP): A climate‐change impact indicator, reported as kg CO_2_‐equivalent per functional unit (e.g., per kg of product). Comparable reference values are *cradl‐to‐gate* GWP of various types of steel: ∼1–4 kg CO_2_‐eq/kg [6], or polylactic acid (PLA): ∼3 kg CO_2_‐eq/kg [7].
**•** Ecotoxicity: Potential of substances emitted over the life cycle (e.g., solvents, heavy metals, degradation products) to harm aquatic and terrestrial organisms, typically quantified via characterization models that translate emissions into toxicity‐related impact indicators (e.g., comparative toxic unit equivalents (CTUe) per functional unit; 1,4‐dichlorobenzene‐equivalent (1,4‐DCB‐eq); or 2,4‐dichlorophenoxyacetic acid‐equivalent (2,4‐D‐eq)).
**•** Process mass intensity (PMI): The ratio of the quantity of raw materials required per kg of product (often corrected for water or impurity content).
**•** Synthetic cell: A concept that refers to life‐like assemblies that aim to recreate living cells. Synthetic cell components are divided into compartment‐forming (e.g., lipids) and encapsulated components (e.g., proteins or cell‐free systems).
**•** Phospholipids: Amphiphilic molecules that self‐assemble into bilayers and vesicles. They form the membrane compartments of most synthetic cells.
**•** Diblock copolymers: Synthetic molecules containing hydrophilic and hydrophobic polymer blocks that self‐assemble into membrane‐like structures. They are often more stable and less permeable than lipid membranes.
**•** Cell‐free protein synthesis (CFPS): In vitro protein synthesis using purified components or cell extracts without living cells. In synthetic cells, CFPS enables the production of proteins such as enzymes and membrane channels.

Components typically used for the bottom‐up assembly of synthetic cells include lipids, diblock copolymers, proteins from recombinant or endogenous sources, and standard buffer and media components. We review available data on the environmental impact of these commonly used materials and on phase‐transfer assembly methods. One quantitative focus of this review is global warming potential (GWP), but we also report other impact categories, particularly ecotoxicity arising from the materials and reagents used. We generally focus on the *cradle‐to‐gate* system boundary, which covers raw material extraction through production to the final product, but excluding end‐of‐life stages such as degradation (*cradle‐to‐grave*). Where available, we compare different process yields and sensitivities to other variable parameters to show the range of reported values. Finally, we benchmark these data against reference values for potential applications and discuss shortcomings of this review, available data, and the LCA methodology in general.

## 2 Methodology

We conducted a literature search using Google Scholar, NCBI, Elicit, and OpenAI ChatGPT 5.1. All cited references were carefully checked and verified. Calculations for the estimation of GWP and freshwater ecotoxicity (FET) impacts for cell‐free protein synthesis (CFPS) reactions prepared from *Escherichia coli*‐derived cell‐free extracts or the OnePot PURE (protein synthesis using recombinant elements) system were performed based on interpolated literature data. Jupyter notebooks underlying the calculations can be found at https://github.com/Bio‐inspired‐Computation‐Lab/syncell_impact.

### 2.1 Environmental Impacts of Lipid Extraction and Synthesis

A well‐established compartment‐forming material for synthetic cells is soy lecithin. Lecithin is a mixture of phospholipids with varying head groups and tails together with additional amphiphiles and minor components, such as glycolipids and carbohydrates. In water, lecithin self‐asssambles into lamellar phases that allow for the formation of giant unilamellar vesicles (GUVs), a major class of synthetic cell compartments. The base soy oil extract used to prepare lecithin has a reported *cradle‐to‐gate* CO_2_ impact in the order of 1 kg CO_2_‐eq/kg and a low toxicity profile [8]. Lecithin extracts are obtained from acetone‐insoluble pellets using a 1:1.5 mixture of water and soy oil. While the low‐purity acetone‐extracted lecithin fraction has already been reported to form cell‐sized vesicles [9], the higher‐phospholipid‐content product typically used in the synthetic cell field requires further purification [10], for which no detailed LCA is available. Clearly, emissions increase with purity and compositional specificity, as one study reporting 125 kg CO_2_‐eq/kg of high‐purity egg phospholipids shows [11]. The most popular lipids in the synthetic cell field (e.g., 1,2‐dioleoyl‐*sn*‐glycero‐3‐phosphocholine (DOPC), 1‐palmitoyl‐2‐oleoyl‐phosphatidylethanolamine (POPE)) are produced via semisynthetic pathways starting from egg/soy lecithin phosphatidylcholine (PC), followed by enzymatic modifications. Due to the additional processing steps involved, their emissions are necessarily higher than those from high‐purity total lipid extracts. Non‐natural lipids (e.g., 1,2‐dioleoyl‐3‐trimethylammonium‐propane (DOTAP)) require fully synthetic precursors, while other specific lipids, such as phosphatidylinositol (PI), must be extracted from mammalian cell culture. To our knowledge, no LCA studies exist for any of these individual lipids.

Extensive LCAs and environmental‐footprint optimizations have been performed for the extraction of lipids from microalgae, primarily in the context of biofuels and food. While the use of algae lipids for synthetic cells is not yet well established, there is evidence that these lipids are suitable for forming cell‐sized compartments [12]. The process of lipid extraction was identified as the main hotspot in the downstream processing of biofuels from microalgae because of high energy investment for cell lysis and the necessary solvent recovery [13]. For example, a process in which total lipids are obtained by cell lysis, centrifugation, and methyl‐*tert*‐butyl ether (MTBE) solvent extraction reported approximately 20 kg CO_2_‐eq/kg of fatty acid product at a site in Tuscany, Italy [14]. Avoiding solvents and their energy‐intensive recovery, for example, by supercritical CO_2_ extraction, has been shown to yield a high‐purity product with reduced emissions [15]. Further extraction methods optimized for environmental impact (e.g., via “green” solvents or mechanical lipid extraction) were studied [16]. The synthetic cell field might learn from these advances to study synthetic cell formation by sustainably sourced lipids.

### 2.2 Environmental Impact of Polymer Synthesis

Poly(ethylene oxide)‐*block*‐poly(butadiene) (PEO‐*b*‐PB) and polyester‐based block copolymers containing polylactic acid (PLA), polyglycolic acid (PGA), or poly(lactic‐*co*‐glycolic) acid (PLGA) are relevant amphiphilic materials for the fabrication of polymersome vesicles in synthetic cell technology. Compared to phospholipid liposomes, whose bilayers are typically about 3–5 nm thick, polymersomes have thicker membranes, in the range of 5–50 nm, with correspondingly higher bending rigidity, greater resistance to rupture, and generally lower passive permeability [17, 18]. However, increased membrane viscosity and mechanical stiffness may also reduce water permeability and complicate membrane protein insertion, although tuning the polymer chemistry or using hybrid lipid/polymer membranes can partly overcome these limitations [19]. In addition, soluble PEO (also known as polyethylene glycol (PEG)) is also an important component to induce molecular crowding, in particular for the formation of coacervates [20, 21].

PEO‐*b*‐PB is usually synthesized by sequential living anionic polymerization. In a typical route, *n*‐butyllithium first initiates the polymerization of 1,3‐butadiene to form the PB block; ethylene oxide is then added and polymerized by ring‐opening to grow the PEO block, often in the presence of strong phosphazene bases [22]. PLA/PGA/PLGA‐based amphiphilic block copolymers are typically synthesized through ring‐opening polymerization of lactide and/or glycolide, often using hydroxyl‐terminated PEG/PEO as a macroinitiator to form PEG‐*b*‐PLA, PEG‐*b*‐PGA, or PEG‐*b*‐PLGA amphiphiles suitable for vesicles [23, 24, 25, 26]. In PLGA, the lactic/glycolic ratio can be used to tune hydrophilicity, crystallinity, mechanical properties, and degradation rate, making PLA/PGA/PLGA systems especially relevant for degradable or transient synthetic cell compartments [25, 27].

To the best of our knowledge, there is no dedicated LCA yet for synthetic‐cell‐grade diblock copolymers, but LCAs of its monomers provide a first estimate of its *cradle‐to‐gate* GWP. 1,3‐Butadiene is mainly obtained as a co‐product of naphtha steam cracking or via dehydrogenation of 1‐butene. The naphtha‐based route was reported to have a GWP of around 2.5 kg CO_2_‐eq/kg 1,3‐butadiene [28]. Alternative bioethanol‐based butadiene routes can lead to lower, higher, or even net‐negative emissions depending on the feedstock used for bioethanol production. However, although bioethanol‐based routes can reduce GWP and fossil resource use relative to naphtha‐based production, they may increase water consumption, energy demand, land‐use‐related impacts, acidification, or eutrophication [28]. Ethylene oxide (PEG/PEO) is produced by silver‐catalyzed epoxidation of ethylene [29]. Conventional ethylene oxide production emits around 2.3 kg CO_2_‐eq/kg, whereas emerging CO_2_‐electrolysis routes could reduce that footprint under favorable electricity and process‐efficiency assumptions [30]. Hydrating ethylene oxide to ethylene glycols appears to add comparatively little to the overall GWP [31]. Combining these monomer‐level proxies suggests that the diblock copolymer falls in the single‐digit kg CO_2_‐eq/kg polymer range; however, this estimate should be interpreted as indicative because it omits diblock copolymer synthesis, solvent use, purification, molecular weight control, and waste treatment.

For PLA/PGA/PLGA materials, a meta‐analysis of more than 80 PLA LCA and carbon footprint studies reported a median *cradle‐to‐gate* GWP of 1.63 kg CO_2_‐eq/kg PLA resin, and 0.5 kg CO_2_‐eq/kg PLA when biogenic CO_2_ uptake was included [32]. LCA data for PGA and PLGA are more limited, but a prospective LCA of CO_2_‐derived PLGA suggests that replacing fossil feedstocks with captured CO_2_ and biomass can reduce greenhouse gas emissions in selected scenarios, depending strongly on electricity supply, CO_2_ source, product application, and end‐of‐life assumptions [33]. Taken together, biomass‐based polymers may offer a more degradable design space than petrol‐based polymers, but they should not be assumed to have lower total environmental impact without polymer‐specific life cycle inventory (LCI) data.

Apart from the synthetic polymers discussed above, oligonucleotides are an important class of structural or sequence‐programmable building blocks for synthetic cells. Their manufacturing is material‐intensive: reported process mass intensities (PMI) span 3035–7023 kg of input per kg of product, with PMI usage roughly split between synthesis and purification [34]. Additionally, the reagent inventory poses environmental challenges, as it includes compounds such as dichloroacetic acid (DCA) and aqueous ammonia [34]. The main solvent, acetonitrile, contributes approximately 1170 kg CO_2_‐eq/kg of oligonucleotide product even under the optimized conditions of solid‐supported synthesis in plug‐flow mode [35]. Alternative solvents such as acetone have been explored to reduce the environmental footprint of oligonucleotide manufacturing in the pharmaceutical industry, but so far, no full replacement for acetonitrile has been demonstrated at scale [34].

### 2.3 Environmental Impacts of Lipid and Polymer Modifications

Functionalized lipids and polymers are often used to give synthetic cell membranes fluorescence, affinity binding, adhesion, permeability control, or stimuli‐responsive behavior. Examples include PEGylated, Ni^2+^‐charged nitrilotriacetic acid (Ni‐NTA)‐, and dye‐modified lipids, as well as polymers with reactive, fluorescent, or stimulus‐responsive groups [36, 37]. However, direct LCA data for these synthetic‐cell‐specific modifications are largely missing. PEG modification of lignin was reported to range from 0.85 to 5.64 kg CO_2_‐eq/kg, depending on the process scale [38]. In particular, for dye modifications, wastewater studies show that synthetic dyes can create aquatic ecotoxicity and health concerns if released untreated [39].

### 2.4 Environmental Impacts of Recombinant Protein Production and Cell‐Free Synthesis

Cell‐free extracts enable synthetic cells to synthesize proteins from linear or plasmid DNA through a combined transcription–translation (TX–TL) reaction. The cellular TX–TL machinery for mRNA and protein synthesis is extracted from a cellular source. A variety of different cell‐free systems exists that can be categorized by the source organism and the purity of the extract components. Here, we focus on two popular cell‐free systems in the synthetic cell context, namely the so‐called crude “S30” *E. coli*‐based cell‐free extract, and the protein synthesis using recombinant elements (PURE) system [40, 41, 42]. While the crude extract is essentially the soluble cytosolic fraction after lysis, the PURE system is assembled from 36 affinity‐purified recombinant proteins.

#### 2.4.1 Recombinant Protein Production

LCA data for recombinant protein production is relatively well studied, and we therefore begin by examining the recombinant production of proteins. The environmental footprint of recombinant protein products varies widely depending on the carbon source, host organism, expression strategy (intracellular vs. secreted), downstream processing, energy supply, yield, and process scale [43]. For example, recombinant‐protein‐based growth factors produced at laboratory scale can have very high GWP intensities per unit mass with estimated values of 0.4–2 × 10^5^ kg CO_2_‐eq/kg [44]. At laboratory scale, electricity dominates (>76% of most impact categories), and the bioreactor stage is typically the single largest contributor, followed by downstream processing (≈30%–40%). Upon scale‐up, the relative burden shifts toward media chemicals (e.g., nitrogen sources), and the per‐unit GWP falls by several orders of magnitude [45]. The use of genetically engineered expression hosts has been reported to reduce emissions by up to sixfold through more efficient use of fermentation feedstock, though the source and pretreatment of fermentation ingredients remain major drivers [43]. Overall, GWPs reported for industrially manufactured enzymes with established processes are in the 1–25 kg CO_2_‐eq/kg range, depending on the functional unit and system boundaries [43, 46]. A recent comparative LCA of six recombinant enzyme production routes reinforces that feedstock and host choices matter: replacing refined glucose with sea lettuce, wheat straw, or phototrophic CO_2_ assimilation reduced fermentation‐related emissions by ∼51%, ∼64%, and ∼80%, respectively. Switching from organic to inorganic nitrogen sources also showed potential to lower marine eutrophication, land use, and ozone depletion burdens. The composition of the local energy mix has a notable impact on the GWP, shifting it by approximately 14%–27% [47]. Importantly, GWP is not the dominant pollutant category in protein manufacturing. Considering normalized results, the human and ecosystem toxicity categories (especially the freshwater and marine ecotoxicity, FET and MET, respectively) can account for approximately 99% of the overall score, with burdens often originating from medium components (notably phosphate), cooling brine, and purification reagents [47]. Downstream affinity chromatography purification represents an additional hotspot. Chromatography (with Tris‐HCl washing/regeneration) increases marine ecotoxicity by 3.4–3.8 times and increases overall impacts by 4.6 times to a peak value of 26 kg 1,4‐DCB‐eq FET [47].

#### 2.4.2 E. Coli‐Based PURE and S30 System

To estimate the environmental impact of a OnePot PURE CFPS reaction produced at an industrial scale, we used LCA data from an industry‐scale production process for recombinant enzymes, including fermentation and purification steps [47], and the protein yield per culture volume of a OnePot PURE expression culture [40] (see supplementary Jupyter notebook, GWP_FET_calculations.ipynb). Based on this, the GWP contribution of the recombinant protein fraction to the environmental impact of 1 L OnePot PURE CFPS reaction would be 129 kg CO_2_‐eq, with an FET value of 3.1 kg 1,4‐DCB‐eq. In comparison, the estimated GWP contribution for the production of *E. coli* cell‐free extract is 2–9 kg CO_2_‐eq/L CFPS reaction with an FET value of 0.1–0.2 kg 1,4‐DCB‐eq. In this scenario, the industrial‐scale recombinant protein production encompasses fermentation and downstream processing steps, the latter contributing about 82% and 50% to the total GWP and FET values, respectively [47]. Since these steps include a protein purification step via immobilized metal affinity chromatography (IMAC), a process not included in S30 cell‐free extract production, the upper and lower boundaries of the GWP and FET estimates for cell‐free extract production are based on the recombinant protein production data, including or omitting the downstream processing steps, respectively. Therefore, it is reasonable to expect the GWP and FET values for 1 L CFPS reaction from cell‐free extract prepared at an industrial scale to fall within the lower end of the estimated ranges.

Beyond the cell‐free extract or the PURE proteins, which account for approximately 17% and 3% of the CFPS reaction by weight, respectively, the necessary bulk components are potassium and magnesium salts, e.g., glutamates, and an optional buffer component, such as HEPES (4‐(2‐hydroxyethyl)‐1‐piperazineethanesulfonic acid). These components typically constitute approximately 48% of an extract‐based CFPS reaction by weight. Monosodium glutamate LCA data can serve as a proxy for magnesium glutamate, one of the main bulk components. The *cradle‐to‐gate* GWP of monosodium glutamate from corn starch in China is calculated to be 4.6 kg CO_2_‐eq/kg [48], with energy consumption and steam usage as the largest contributors across the extraction, refinement, and fermentation steps [49]. In addition, the production and refinement process requires maize, ammonia, sulfuric acid, and potassium hydroxide as main raw materials, and produces large quantities of wastewater [50]. The final CFPS reaction is assembled by supplementing the reaction mixture with amino acids, nucleoside triphosphates (NTPs), an energy regeneration system, and additional minor components with an overall mass fraction of approximately 35% of an extract‐based CFPS reaction. Here, certain components may drive substantial emissions through multistep purification and functionalization processes, and should be prioritized when optimizing for environmental impact. For example, NTPs are preferably produced by fermentation with genetically engineered microbial strains (e.g., *E. coli*, *Bacillus subtilis*, *Corynebacterium glutamicum*, yeast) that overproduce the corresponding nucleotides, nucleosides, or monophosphates. Nucleosides and monophosphates are subsequently enzymatically phosphorylated by specific kinases (ATP [51], CTP [52], GTP [53, 54], UTP [55]), and the resulting NTPs are finally purified, e.g., by ion‐exchange chromatography and/or crystallization [56]. Although there is no LCA data available for NTP production, data for a similar process, the production of 2′3′‐cyclic GMP‐AMP via fermentation and subsequent enzymatic cyclization of GTP and ATP, calculate the GWP of the production of ATP and GTP substrates to be 1253 kg CO_2_‐eq/kg [57], with long fermentation times and elaborate purification having the highest impact.

### 2.5 Environmental Impacts of Synthetic Cell Assembly Methods

It is interesting to note that the assembly of synthetic cells is an energetically unfavorable process that requires a driving force. While in principle, light mechanical agitation of a lipid film in water is sufficient to form lipid‐based synthetic cell compartments, the methods that promise efficient high‐throughput assembly are based on two‐phase emulsions of water and lipid–oil mixtures. The rather large volumes of lipid–oil mixture are a potential source of emissions. Manufacturers report GWP for commonly used silicone oil in the 2–6 kg CO_2_‐eq/kg range [58, 59]. Fluorinated oils (e.g., FC‐40 or HFE‐7100) and fluorinated surfactants (e.g., perfluoropolyether (PFPE)‐based block copolymers) have seen increased use in synthetic cell production due to high emulsion stability and other advantageous properties [60]. Generally, while these second‐generation fluorinated products are intended to replace hydrofluorocarbons and ozone‐depleting substances, they are still compounds with high global warming potential. Focusing on production, fluorinated oils are obtained via energy‐intensive electrochemical fluorination and may emit lower‐molecular‐weight fluorinated or other halogenated organic molecules, adding to the overall footprint, which is not yet fully understood [61]. Thus, the reported value of 21 kg CO_2_‐eq/kg for structurally related polytetrafluoroethylene (PTFE) [62] might not fully account for the total impact. Indeed, GWP values in the range of 10^2^–10^3^ kg CO_2_‐eq/kg have been reported for the synthesis of the fluorinated compounds C_7_H_7_F and C_7_H_5_F_3_O, but these should not be directly taken as a proxy for fluorinated oils due to differences in the synthesis route.

Taken together, the production and purification of oil might add a substantial burden because of the excess oil needed to form a single compartment. Therefore, it is interesting to note the variation in efficiency between bulk two‐phase and microfluidic systems. For example, a yield of approximately 20,000 lipid‐bound 25 µm‐diameter compartments for a concentration of 50 µM lipid in a 50 µL lipid–oil mixture was reported in a bulk phase‐transfer process [63]. This corresponds to an excess oil usage of nanoliters per synthetic cell compartment, which is up to 10^5^ times more than the small solvent droplets (in the upper femtoliter range) that separate during microfluidic chip production [64]. Notably, the fluorinated oil FC‐40 has been used in both bulk and microfluidic approaches [60, 65]. The excess oil consumption in these two systems should be compared to the picoliter volume of a synthetic cell and shows that the excess oil amount needed varies between 0.1–1000 v/v for different preparation conditions. Overall, these numbers demonstrate the high potential of recycling lipid‐oil mixtures for synthetic cell formation, but we are not yet aware of a demonstration of such a process.

For the microfluidic device itself, LCAs show that the environmental burden per device is dominated by polymer production and clean room energy use [66]. Microfluidic devices are often assembled from a type of cross‐linked silicone oil, polydimethylsiloxane (PDMS), adding to the overall amount of oil used per compartment. Toxicity impacts for microfluidic fabrication stem mainly from the use of solvents and photoresists [66, 67]. Thus, the lifetime of a single microfluidic device would be a major determinant in assessing the environmental impact. Since large‐scale production data on synthetic cells are not yet available, the contribution of the microfluidic device technology cannot currently be quantified, but it could be significant.

### 2.6 Cell‐Free Extracts and Phase‐Transfer Production Dominate Synthetic Cell Formulation's GWP

**FIGURE 2:**
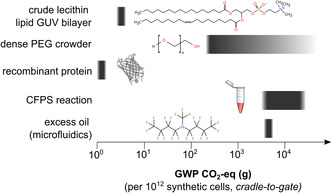
Selected feedstock mass of synthetic cell components and their GWP for 10^12^ synthetic cells, *cradle‐to‐gate*, with values from Table 2.

In Table 2 and Figure 2, we compare the indicative GWP (CO_2_‐eq) across the main impact categories of synthetic cell components for a functional unit of 10^12^ synthetic cells of 15 µm diameter. To contextualize these values, we compare them to the CO_2_ fixation capacity of the crotonyl‐CoA/ethylmalonyl‐CoA/hydroxybutyryl‐CoA (CETCH) cycle. This artificial carbon fixation cycle was demonstrated in synthetic‐cell‐like compartments, relying on a plant thylakoid membrane extract and a set of recombinant proteins, with a reported (unoptimized) fixation rate of approximately 47 µM glycolate from CO_2_ over 90 min [68]. This translates to a CO_2_ fixation rate of 2.8 mg CO_2_/L per h. Scaling this rate to our functional unit of 10^12^ synthetic cell compartments with a total volume of 1.8 L suggests that it could fix 4.9 mg CO_2_ per h (118 mg per day; 0.8 g per week, 3.5 g per month), meaning such systems could offset the GWP of the recombinant components within weeks to months of continuous operation. It should be noted that such long continuous operation is not realized yet. However we want to empathize another point, which is that the discussed CETCH system relied on fluorinated oil to stabilize compartments, a component with substantial GWP that would move net carbon negativity completely out of reach. This comparison is therefore partly encouraging, but also illustrates how early design choices can strongly influence the overall environmental impact.

**TABLE 2:** Selected feedstock mass of synthetic cell components and their GWP for 10^12^ synthetic cells *cradle‐to‐gate.* Some values are aggregated from nonstandardized sources and therefore have large uncertainties (see main text for detailed discussion).

Synthetic cell component	Dry mass/volume	GWP CO_2_‐eq
Crude lecithin lipid GUV bilayer^a^	3 g	3 g
Dense PEG crowder (100 mg/mL)^b^	180 g	>400 g
1 µM generic recombinant protein^c^	0.081 g	0.81 g
CFPS reaction (contribution from *E. coli* cell‐free extract only)^d^	1.8 L	3600–16,200 g
Microfluidic production (excess fluorinated FC‐40)^e^	0.18 L	>4995 g

a Assuming an area per lipid of 0.67 nm^2^ and lipid molecular weight 810 g/mol.

b Using the value of 2.3 CO_2_‐eq/kg of EO synthesis as a lower bound.

c Assuming a typical value of 10 kg CO_2_‐eq/kg for an optimized industrial process and a MW of 45 kDa.

d 2–9 kg CO_2_‐eq/L CFPS reaction, depending on extract postprocessing.

e Assuming a conservative estimate of 15 CO_2_‐eq/kg and 0.1 v/v excess oil per compartment without recycling of lipid–oil mixture.

The values in Table 2 can also be set in the context of pharmaceutical manufacturing, where a model compound, synthesized from cyclohexanone and cyanoacetic acid in several steps and racemate‐purified, exhibited a GWP of 67 kg CO_2_‐eq/kg product [69]. Depending on formulation, synthetic‐cell‐based technology for drug delivery could therefore contribute substantially to the overall environmental footprint of a pharmaceutical product.

Looking further ahead, autonomously replicating bottom‐up synthetic cells are unlikely to improve on these figures in the near term. A primitive synthetic cell would probably still rely on inefficient energy metabolism and high‐energy exogenous compounds, both of which would add to emissions. This is already a known challenge encountered in cultured meat production, where the composition of mammalian cell culture media is a major driver of the environmental footprint [70].

### 2.7 This Literature Study and System Boundaries

For this work, we identified major synthetic cell components and conducted a literature search for related LCA studies focusing on *cradle‐to‐gate* production. LCAs are known to vary widely between studies, with order‐of‐magnitude differences across impact categories. These discrepancies typically stem from inconsistencies in the emission values included in the inventory, the substances considered, and the characterization factors applied to those substances [71]. In addition, allocation choices, physical location of a process, and system boundaries play a major role [72, 73]. We have often encountered incomplete or not easily accessible LCI data, which poses a significant challenge for advancing sustainable research in the field of synthetic cells. Although curated databases provide some LCI data, these are often not openly accessible. We were further limited by data availability and incomplete reporting for complex multistep processes such as fermentation and cell‐free extract production, where the full process could not be accounted for. As a result, the values reported here for CFPS may underestimate total emissions, but could equally overestimate them, since potential synergistic effects and more efficient use of extracted material were not considered. For example, using lipids extracted from *E. coli* fermentation during cell‐free extract preparation could lower the overall impact. In addition, feedstock selection and the use of coproducts can significantly reduce emissions, and, in principle, even enable net carbon removal. Membrane‐forming lipids, for example, can be extracted from a wide range of organisms, offering flexibility in selecting particularly suitable hosts. Therefore, the component‐level data reviewed here cannot simply be summed to estimate the full environmental impact of a synthetic cell; rather, it is intended as a starting point for a comprehensive LCA of synthetic cell production.

While our review focuses on *cradle‐to‐gate* data, we briefly address some end‐of‐life aspects here. Notably, fluoropolymers used in microfluidic production are considered part of the class of per‐ and polyfluoroalkyl substances (PFAS) [61], with GWP values of around 320 kg CO_2_‐eq/kg over a time horizon of 100 years [74]. In general, synthetic polymers tend to be more persistent than lipids extracted from natural sources, which are expected to degrade fully in the environment. In diblock copolymers, the hydrophobic PB block is thought to degrade slowly via oxidative scission, yielding smaller, potentially persistent fragments in the environment [75]. While the hydrophilic PEO (PEG) block may show low acute aquatic toxicity and low bioaccumulation, water‐soluble synthetic polymers in general are still a concern when released into the environment [76, 77, 78]. Overall, high‐molar‐mass polymers are increasingly recognized as a data‐poor but potentially persistent class, and current assessment frameworks call for more systematic fate and ecotoxicity testing [77, 79].

## 3 Conclusion and Future Research Directions

In this review, we have compared LCA and inventory data for various feedstocks and processes relevant to synthetic cell production. We were surprised by the relatively high emissions associated with the use of *E. coli* extract, driven by energy‐intensive fermentation, preparation, transport of media components, and cell lysis. Emissions from the PURE system may even be higher, underscoring the need for more detailed studies. Compared to these emissions, contributions of individual recombinant proteins and low‐purity lipids seem negligible. However, as noted recently, current synthetic cells are much more dilute than living cells, indicating that certain burdens might still rise in the future [80]. Also notable is the large excess of material and the potentially concerning use of fluorinated compounds in synthetic cell production. In addition, a clear trend observed in this literature study is the increased environmental impact of high‐purity products, where, for example, additional processing steps after cell lysis contribute substantially to emissions. In contrast, crude‐lipid extracts based on acetone extraction have a very low environmental footprint, and acetone synthesis was demonstrated via a carbon‐negative industrial‐scale process [81], indicating potential synergistic effects with ongoing efforts to transition toward more sustainable commodity chemicals. This suggests that future work should also focus on minimum purity requirements for individual components and consider copurifying multiple components from the same source. Going further, sourcing synthetic cell components from autotrophic organisms could reduce or eliminate dependence on fermentation‐based growth. While creating new LCI and LCA data should be an important aspect of future research, it is equally important that this data is openly accessible, ideally in a curated and standardized form. Since there is no “natural home” to this kind of data yet, a specialized community‐maintained repository based on the FAIR data principle (findable, accessible, interoperable, reusable) could be a useful addition to other community‐driven approaches in the synthetic cell field. Finally, our study underscores that early design choices in synthetic cell development will critically shape future scale‐up pathways and environmental viability.

## Funding

This study was supported by Bundesministerium für Bildung und Forschung (031B1533A), and European Research Council (101163768).

## Conflicts of Interest

The authors declare no conflicts of interest.

## Data Availability

The data that support the findings of this study are openly available in Bioinspired Computation Lab Github at https://github.com/Bio‐inspired‐Computation‐Lab/syncell_impact.
